# Capturing continuous, long timescale behavioral changes in *Drosophila melanogaster* postural data

**DOI:** 10.1371/journal.pcbi.1012753

**Published:** 2025-02-03

**Authors:** Grace C. McKenzie-Smith, Scott W. Wolf, Julien F. Ayroles, Joshua W. Shaevitz

**Affiliations:** 1 Department of Physics, Princeton University, Princeton, New Jersey, United States of America; 2 Department of Physics, Wesleyan University, Middletown, Connecticut, United States of America; 3 Lewis-Sigler Institute for Integrative Genomics, Princeton University, Princeton, New Jersey, United States of America; 4 Department of Ecology and Evolutionary Biology, Princeton University, Princeton, New Jersey, United States of America; Duke University, UNITED STATES OF AMERICA

## Abstract

Animal behavior spans many timescales, from short, seconds-scale actions to daily rhythms over many hours to life-long changes during aging. To access longer timescales of behavior, we continuously recorded individual *Drosophila melanogaster* at 100 frames per second for up to 7 days at a time in featureless arenas on sucrose-agarose media. We use the deep learning framework SLEAP to produce a full-body postural dataset for 47 individuals resulting in nearly 2 billion pose instances. We identify stereotyped behaviors such as grooming, proboscis extension, and locomotion and use the resulting ethograms to explore how the flies’ behavior varies across time of day and days in the experiment. We find distinct daily patterns in all stereotyped behaviors, adding specific information about trends in different grooming modalities, proboscis extension duration, and locomotion speed to what is known about the *D. melanogaster* circadian cycle. Using our holistic measurements of behavior, we find that the hour after dawn is a unique time point in the flies’ daily pattern of behavior, and that the behavioral composition of this hour tracks well with other indicators of health such as locomotion speed and the fraction of time spend moving vs. resting. The method, data, and analysis presented here give us a new and clearer picture of *D. melanogaster* behavior across timescales, revealing novel features that hint at unexplored underlying biological mechanisms.

## Author summary

Animal behavior shows patterns across many different timescales, from repeated grooming sequences that occurs over minutes, to the circadian rhythms that encompass a 24-h period, to the changes in behavior that occur over the course of a lifetime. Recent advances in computer vision and machine learning now allow us to measure these behaviors quantitatively, giving us deeper insight into exactly what behaviors are present and the intricate time sequences they form. Here, we measure the continuous behavior of fruit flies over a period of up to 7 days. Our fine-grained analysis allows us to observe new facets of the circadian cycle of behavior, such as daily patterns in the amount of time spent grooming and the speed of locomotion. We find that the well known morning and evening spikes in overall activity levels comprise different sets of behaviors, meaning that they may serve distinct biological purposes. The method we develop for continuous, high-resolution monitoring of behavior can be utilized in future studies to examine how these behaviors are related to longevity and health.

## Introduction

Uncovering the temporal structure of behavior has long been a topic of theoretical interest and experimental challenge [1–4]. Animals carry out sequences of behaviors on many timescales, from the short timescales of the individual movements required for grooming, eating, and social communication to the longer timescales of hunger, arousal, diurnal cycles, mating seasons, and the aging process. The specifics of these behavior sequences determine much of what we can characterize about an animal, such as its health, reproductive fitness, and that idiosyncrasy of action that we might call ‘personality.’ These behavior sequences also give us indirect ways to assess the internal processes of the animal, such as neural activity, gene expression, and other internal states like hunger or fatigue.

An important aspect of animal behavior is how different timescales interact, from hours to days to lifespans. Investigating these interactions requires a model organism with a broad variety of behaviors but a short lifespan, to allow us to capture the breadth of all the timescales it might inhabit. *Drosophila melanogaster* have a rich set of behaviors, many of which are spontaneous and can occur in simple, isolated environments. Over the course of a 24-h light/dark cycle *D. melanogaster* have varying activity levels across hours, with particular peaks of activity in the morning and evening [5,6] that are controlled by distinct populations of neurons [7]. As *D. melanogaster* age their circadian cycles break down [8], and their activity over different hours loses a defined pattern. Investigating the precise details of how the behaviors of hours become the behaviors of days become the behaviors of lifespans requires high-resolution data that covers the many timescales over which animal behavior varies.

Historically, taking long-timescale data covering days or weeks of an animal’s life has required balancing continuity, throughput, and dimensionality. In *D. melanogaster*, simple experimental setups, such as beam-break assays allow for continuous monitoring of activity levels over days [9,10], but fail to capture the high-resolution data necessary for modern techniques of behavior analysis such as MotionMapper [11], B-SOiD [12], VAME [13], or Keypoint-MoSeq [14]. On the other hand, the acquisition of high-resolution data has been restricted to short timescales by the computational resources required to store and process the extremely large imaging data, imposing an upper limit on the order of an hour. When studying fine-grained behavioral variation at longer timescales, previous work utilized short high-resolution recordings taken from different individuals with ages distributed across the lifespan of the animal and unsupervised behavioral classifications [15]. Other studies have taken continuous, lower frame-rate, lower-resolution recordings, which allow for supervised behavioral classifications [16–18]. These studies are able to provide longitudinal behavioral data, but are limited in their ability to identify discrete behavioral states, generally adding an additional non-locomotion/non-idle state which is labeled as “grooming” or “micromovement”.

Here, we leverage recent computational advances to record a high-resolution continuous dataset of *D. melanogaster* behavior spanning 4-7 days. We recorded 47 freely moving *D. melanogaster* using constant IR illumination and an IR-sensitive camera at a frame rate of 100Hz, with a 12-h visible-light day/night cycle. We tracked 14 body parts from each fly using SLEAP [19] and utilized MotionMapper [11], an unsupervised machine-learning method, to develop data-driven definitions of stereotyped behavioral states, such as grooming, locomoting, and proboscis extension. These definitions are able to discriminate between different grooming modalities (such as wing grooming vs. abdominal grooming), and have a temporal resolution that can quantify individual steps.

We use techniques of compositional data analysis [20] to characterize the dynamics of this behavioral repertoire across time of day and over the days of the experiment. We recapitulate many previously known characteristics of the *D. melanogaster* circadian cycle, and find distinct daily patterns in all measured behaviors, including grooming, proboscis extension, and locomotion speed. We see an overall decline in the difference in behavior between day and night hours across days in the experiment as flies weaken and die, similar to the known weakening of circadian rhythms with age. Additionally, we find a general decline in proboscis extension and locomotion speed as the fraction of time spent in an idle state increases across days. We identify the hour after dawn as ethologically unique, and show that this time point appears to track the general behavioral health of the flies. Our high-resolution data also reveals a difference in behavioral patterns during the morning and evening peaks of activity, primarily appearing in the grooming component of behavior. Overall, we find that our data captures both expected and novel patterns of *D. melanogaster* behavior across multiple 24-h periods. Our findings, particularly with regards to the ethological distinction between the morning and evening, offer a new window into the hourly and daily patterns of *D. melanogaster* activity, and represent a significant step forward in our ability to quantify the inherently multi-timescale structure of animal behavior.

## Results and discussion

### Continuous recording of high resolution behavior data

**Fig 1:**
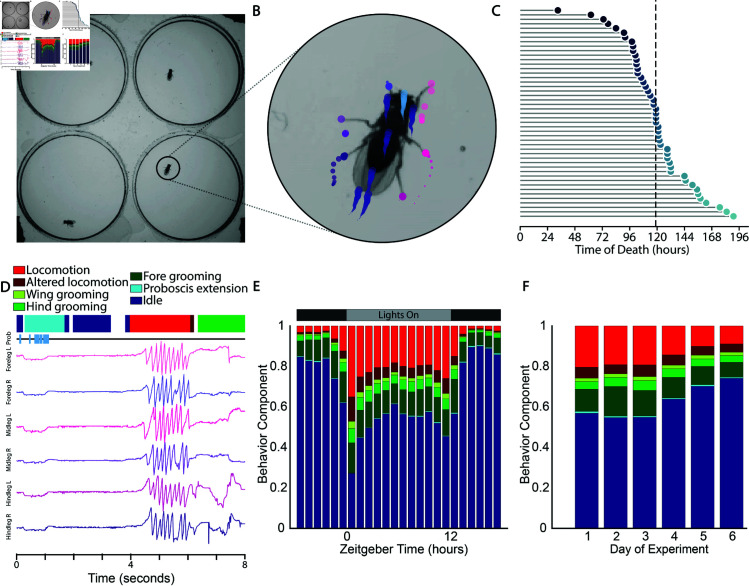
Experimental schematic showing tracking, lifespans, and behavioral segmentations across timescales. **A** Image showing the experimental arena as viewed from below. The behavior of 4 *D. melanogaster* is captured simultaneously while giving each fly enough room to freely carry out all behaviors except flight. **B** Magnified view of a single individual showing tracks for each node of the SLEAP skeleton. Each color denotes a node and circle sizes increase with time. **C** Survival curve of the 47 flies included in the experiment. Death occurs on average after  ~ 119 h, or almost 5 full days into the experiment. **D** Ethogram and egocentrized traces for each tarsi and a raster denoting proboscis visibility. **E** Barplot showing the geometric means of stereotyped behavior components across all flies and all complete 24 h periods. **F** Barplot showing the geometric means of stereotyped behavior components across all flies and all hours grouped by experimental day.

We designed a recording apparatus to allow for continuous capture of *D. melanogaster* behavior over the course of days (see Methods for details and S1A Fig). *D. melanogaster* were constantly illuminated from above with IR light, to which they have minimal visual sensitivity [21], while LED panels provided a 12-h visible-light day/night cycle. Flies lived and behaved in 25 mm diameter, 1.5 mm high cylindrical arenas over the course of our experiments (S1B Fig). We provided the flies with a base gel layer of sucrose-agarose, which permitted survival of up to 7 days while preventing the significant fungal growth observed when yeast extract was included. We recorded four freely behaving *D. melanogaster* in individual chambers per camera at 100 Hz with a resolution of 28.25 pixels/mm (Fig 1A). This is sufficient to resolve relevant features of the *D. melanogaster* body such as the tarsi (leg tips) and proboscis (Fig 1B).

Our experiments have both a change in light intensity and temperature conditions between day and night, with daytime temperature levels varying between different experiments ( ~ 28–29 ∘C for experiments 1–2, and  ~  30–31 ∘C for experiments 3–4) and nighttime temperatures settling to  ~ 27 ∘C for all experiments (see Methods and S9 Fig). These unusually high temperatures are primarily due to our intense IR back light, which facilitated a very short exposure time for low motion blur imaging. It is important to note that heat shock proteins can be activated at these temperatures and temperatures in this range influence lifespan [22]. Our results represent the combined effect of light intensity and temperature changes over the course of a day.

For each experiment, we imaged male ${\text{iso}}^{KH11}$ *D. melanogaster* from two days post-eclosion (emergence from the pupa as an adult insect) until death, yielding 4-8 days of continuous recording with half the flies dying by Day 5 (Fig 1C), for a total of 5,584 fly-hours. Note that this lifespan is shorter than conventional assays, likely due to the nutrient-limited sucrose-based food source used to reduce fungal growth [23] and higher-than-optimal temperatures.

To extract postural information from our data, we used SLEAP, a deep-learning-based framework that can infer animal posture based on user-generated training data [19] (See Methods for details). We tracked a 14-point skeleton comprised of the head, eyes, thorax, abdomen, wing tips, tarsi, and proboscis of each individual (Figs 1B and S2). We limited our subsequent behavior analysis to time points when the flies were on the bottom of the arena (as opposed to the walls or ceiling), as this gave the highest quality tracks (S3 Fig).

In order to quantify discrete, stereotyped behaviors, we modified the MotionMapper pipeline [11] to parallelize more steps and optimize use for postural data instead of raw images (S4 Fig). We identified seven well-defined behaviors: idle, proboscis extension, fore grooming (of the eyes or forelegs), hind-grooming (of the abdomen or hindlegs), wing grooming, locomotion, and altered locomotion (often involving slipping or limping). In addition to these well defined behaviors,  ~ 15% of all time points represent unstereotyped postural dynamics, which we exclude from later analyses. The remaining seven well-defined behaviors form a subcomposition which can be analyzed in a compositional data analysis framework [20].

Because we are using limb tracking rather than general peripheral or sub-locomotion levels of movement as in other pipelines [16,17], we are able to distinguish different types of grooming and proboscis extension as unique behavioral categories, and dis-aggregate true grooming bouts from other sources of sub-locomotion levels of movement such as twitching, fidgeting, and single steps. The resulting ethograms permit analysis of patterns in locomotion, proboscis extension, and grooming (Fig 1D). Because our data is continuous over multiple 24-h periods, we can look at how behavior varies with time of day and across days of the experiment.

### Daily and lifelong patterns of behavior

Our data is closed (i.e. the fraction of time spent in all behaviors must add up to one) requiring us to use methods of compositional data analysis to examine changes across flies, hours, and days [20,24]. Averages of closed data are best calculated as geometric means, which we denote as ‘behavior components’. To discuss daily patterns of behavior, we use Zeitgeber time (ZT), where time is measured from the onset of a periodic stimulus rather than from midnight on a clock, to capture the cyclic nature of diurnal effects. For this set of experiments, ZT = 0 h corresponds to the visible lights coming on, and ZT = 12 h corresponds to the lights turning off.

Looking across all fly hours and all days, we see a distinct diurnal pattern of behavior with higher levels of idle during the dark hours, and more locomotion and grooming during the light hours (Fig 1E). In addition to this general trend in activity levels, our high resolution data reveals that grooming behaviors also follow a daily pattern. In particular, we find that hind and wing grooming make up a significant portion of day-time grooming behaviors, but are almost eliminated during dark hours, suggesting that these grooming modalities may serve a different purpose and/or be under different circadian regulation than fore grooming, which occurs at comparatively larger levels during the day and night hours.

The flies in our experiment lack the strong ‘siesta’ (low activity levels midday) that other work has found. This may be because of the elevated day-time temperature of our experiment, which adds a temperature cycling component as well as a light cycling component to the daily pattern of stimuli, since flies under naturalistic temperature and light cycling exhibit an afternoon peak of activity, rather than a siesta [25]. An additional factor that may be exaggerating this effect is the high-resolution nature of our behavioral data. Previous work has found an elevation of ‘micromovements’ during day-time hours [17]. These movements would not be detectable in most activity-count based systems and would be classified as ‘idle’, but in our experiment are quantified as grooming, proboscis extension, and very short bouts of locomotion including single steps.

The flies exhibit peaks of activity around lights on and lights off, although the morning peak is much more prominent than the evening peak. The first hour after the lights turn on is particularly distinct from all other hours, with higher levels of locomotion and grooming. The locomotion and grooming behavior components increase in the hours leading up to lights on and lights off, indicating the anticipation of the change in lighting condition characteristic of circadian rhythms.

Over the course of the experiment, the flies’ behaviors start changing significantly after Day 3 (Fig 1F). Time spent in idle increases over Days 4 through 6 as flies begin dying due to the sub-optimal environmental conditions and the nutritionally incomplete food of this experiment. Interestingly, while the ratios of the three different grooming modalities remain relatively stable, the fraction of time in altered locomotion compared to locomotion increases. As the altered locomotion state contains bouts of slipping, tripping, and limping, this may indicate age- or stress-associated locomotion deficits. Future studies exploring aging or stress in controlled environmental conditions should consider the ratio of these two behaviors as a potential reporter of health.

### A principal components analysis describes day/night differences in behavior

**Fig 2:**
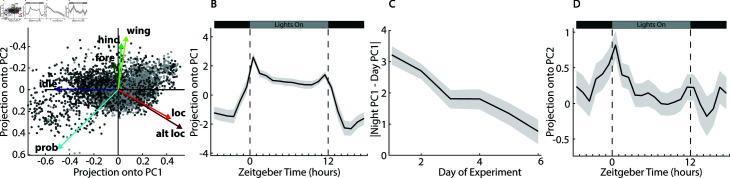
Principal component analysis of stereotyped behavioral components of all flies across all experimental hours. **A** Biplot showing the projections of individual fly hours and loadings of the centered log-ratio of each stereotyped behavioral component. Dark gray dots show time points from when lights are off and light gray dots show time points when lights are on. **B** Mean projection of PC1 against time of day for all complete 24h periods of all flies. The shaded region is the standard error. **C** Mean Day/Night difference vs day of experiment as measured by the absolute value the average projection onto PC1 during night hours minus the projection onto PC1 during day hours. The shaded region is the standard error. **D** Mean projection of PC2 against time of day for all complete 24h periods of all flies. The shaded region is the standard error.

To examine overall behavior variation across hours of the day, we carried out a principal components analysis of the compositional data [26,27] using the compositions package in R [28]. The first two principal components (PCs) explain  ~ 75% of the variance across all fly hours (S6 Fig). As can be seen in the biplot of the first two PCs (Fig 2A), PC1 largely weights the locomotion behaviors (locomotion and altered locomotion) against idle and proboscis extension. This PC describes the main differences between day and night, with positive projection averages during the day, corresponding to more locomotion, and negative values at night when the animals are idle (Fig 2B). The average projection along PC1 begins to increase before the lights turn on, indicating circadian anticipation of the change in lighting conditions. Peak amplitude along this PC occurs just after the lights turn on, potentially indicating a slow morning transition from nighttime to daytime activity. The level of this projection stays roughly constant throughout the day, but then increases and peaks just before the lights turn off at 12h ZT. This is followed by a slow decline in the amplitude after dark until reaching a steady night level.

Previous behavioral studies of the *D. melanogaster* diurnal cycle have used relatively coarse metrics, such as the activity counts generated by *Drosophila* Activity Monitors [9]. These studies have shown that *D. melanogaster* have peaks of locomotion activity around their subjective morning and evening, with the increase in activity slightly anticipating the actual change in lighting conditions [5,6]. Our high-resolution behavioral data and the projection along PC1 recapitulate these general trends, but show qualitative differences when the lights change. In particular, we see a gradual change in amplitude after lights turn off that last several hours whereas this previous work sees a more abrupt cessation of activity at this time [5]. It should be noted that while PC1 is primarily weighting locomotion vs. idle behavior, it does contain grooming and proboscis extension information, and thus offers a more complete representation of the day/night pattern of *D. melanogaster* behavior than activity counts alone.

### Day/night behavioral differences lessen as flies weaken and die

Since the amplitude along PC1 largely follows the day-night cycle and describes the daily change in behaviors, we use the difference between the average value of PC1 during light and dark hours to measure the strength of the circadian cycle. We find that the strength of this signal decreases steadily over days in the experiment (Fig 2C). Previous studies have found that the sleep/wake cycles of behavior in *D. melanogaster* weaken as they age [8]. While the flies in our experiment were all comparatively young (all died before 10 full days, whereas life expectancy is 2–3 months under ideal conditions), they were living in sub-optimal conditions of relatively high temperature, low humidity, and poor nutrient availability. The gradual weakening over the course of the experiment is in some ways similar to an accelerated aging, and the steady decline in the strength of the diurnal signal over 6 days is similar to the decline in the strength of the sleep/wake cycle seen in other experiments over 60 days [8].

It is notable that the loss of difference between day and night behavior cycles begins immediately, whereas a significant change in the overall behavior profiles does not occur until Day 4 (Fig 1F). In this case, it appears that the strength of the daily behavioral pattern is an earlier indicator of age- or stress-related decline than day-averaged metrics such as overall activity levels or sleep fractions. Much research has shown a profound connection between circadian rhythms and aging [8,29–31]. Future work is needed to examine whether this daily pattern of behavior can be used as an indicator of health and/or resilience.

### Principal component 2 describes the hour after lights on

The second principal component weights the three grooming behaviors (fore, hind, and wing) against the locomotion behaviors and proboscis extension. In particular, the hind and wing grooming modalities that are strongly attenuated during night hours are highly weighted in PC2. The average projection onto PC2 has a distinct peak during the hour just after lights turn on, separating this unique part of the diurnal pattern from the more general day vs. night changes in behavior picked up by PC1 (Fig 2D). This projection shows a pre-stimulus anticipatory rise preceding lights on, and then a gradual decline after the first hour of the day. As with PC1, the pre-dawn rise may indicate circadian anticipation, implying that the distinct behaviors of the hour just after dawn are driven internally, rather than appearing as a reaction to the light stimulus.

### Day 1 flies show a strong diurnal behavior pattern with a unique hour after dawn

We leveraged our high-resolution behavior data to carry out an in-depth analysis of the daily pattern of *D. melanogaster* behavior, focusing on the first day of the experiment when the diurnal cycle was strongest (Fig 2C). Flies were reared from embryos to two day old adults with the same light/dark cycle time and phase as the experiments. Even before enclosing, *D. melanogaster* exhibit daily patterns of certain behaviors, such as larval negative phototaxis [34] and eclosion [35], so it is unsurprising that even 2–3 day old adults already have a strong diurnal pattern.

**Fig 3:**
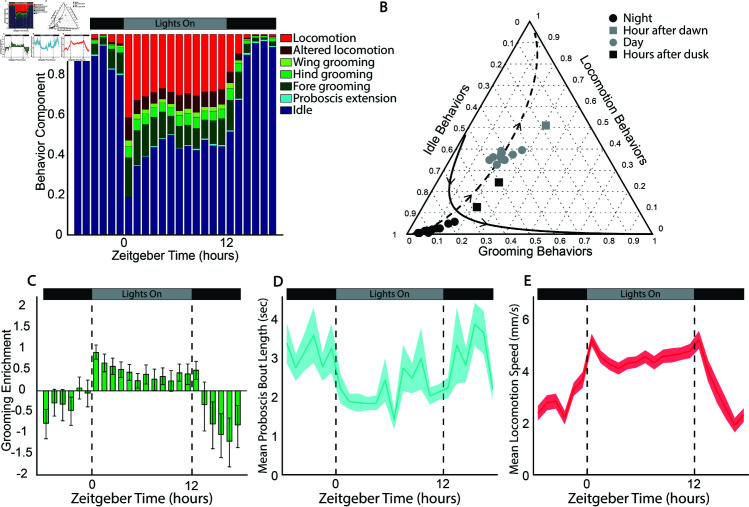
Diurnal patterns of behavior on experimental Day 1. **A** Barplot showing the geometric means of stereotyped behavioral components of the first experimental day across all flies. **B** Ternary plot showing the geometric means of condensed behavioral components across all flies for each hour of Day 1. Directions along PC1 (dashed) and PC2 (solid) as calculated by perturbing the geometric mean of the displayed data points [32]. The ternary plot was generated using the Ternary Plots package in MATLAB [33]. **C** Grooming enrichment with respect to the geometric mean of the condensed grooming behavioral component of the first experimental day for each fly with bootstrapped confidence intervals. **D** Mean proboscis bout length by hour of the first experimental day. The shaded region is the standard error. **E** Mean locomotion speed (mm/s) during stereotyped locomotion state by hour of the first experimental day. The shaded region is the standard error.

The geometric means of behavior components across all flies versus ZT for Day 1 show the expected pattern of increased idle during the night and increased locomotion during the day (Fig 3A). The hour just after lights on remains the most distinct, with a very low idle behavior component. In the evening after lights off, the flies take  ~ 2 h to settle into their characteristic high idle, low locomotion night state. Flies on Day 1 show little in the way of a siesta, and no noticeable evening peak of activity.

The temporal patterns we observe in the two locomotion behaviors (altered locomotion and locomotion) are similar to each other, as are the patterns in the different grooming behaviors (fore, hind, and wing grooming). Additionally, principle components 1 and 2 weight the locomotion behaviors similarly to each other, and the grooming behaviors similarly to each other, as can be seen in the PC space biplot (Fig 2A). Using these strong correlations, we condensed our seven stereotyped behavior components into three categories, grouping together the grooming behaviors, the locomotion behaviors, and idle and proboscis extension. This allowed us to plot the average behavior composition for each hour in a ternary plot to visualize differences in overall behavior across time and along the previously identified PCs (Fig 3B). The day and night hours cluster together and largely lie along PC1 as expected. The 2 h just after lights off fall between these clusters as the flies transition into their night state of behavior. The hour just after lights on is an outlier, falling well away from the line of variance explained by PC1, with higher proportions of grooming and locomotion behaviors compared to all other hours. This hour lies in the direction of increasing PC2, which explains  ~ 17% of the variance across all fly-hours. This, combined with the peak in the projection of behavior components along PC2 during the hour after lights on (Fig 3C), indicates that this hour is a unique time point in the daily cycle of behavior.

Our results here differ from a prior study, which found no particular increase in ‘micromovements’ in the mornings compared to other time points [17]. Because our behavior classifications occur at a higher time-resolution and are based on limb tracking, we are able to observe the pattern of ‘true grooming’, separate from small fidgets and steps.

*D. melanogaster’s* typical morning and evening peaks of activity are regulated by different populations of neurons [7,36]. Certain life stage- or context- dependent behaviors such as eclosion [35] and egg-laying [37] occur in the morning and evening, respectively. We have found that the morning and evening activity peaks are also ethologically distinct in the set of spontaneous behaviors that occur throughout the entire *D. melanogaster* lifespan. In particular, *D. melanogaster* spend the hour after dawn in a high locomotion and grooming state that is not seen in the dusk hours. The difference in both control mechanism and behavioral output may mean that the morning and evening peaks of activity fulfill different biological functions, and could serve as indicators for different aspects of the underlying state of the organism.

### Grooming behaviors are enriched during the day, particularly in the morning

To further investigate the diurnal pattern of grooming, we looked at the enrichment of grooming behaviors at each hour compared to the geometric mean of the grooming behavior component across all hours (Fig 3C). It has been shown that spontaneous grooming is under circadian control [16], but no clear pattern of when grooming happens throughout the day has been identified. We find that grooming behaviors peak in the hour after lights on, contributing to the uniqueness of that time point, in agreement with our analysis of PC2. Grooming serves specific biological functions, such as disease defense and parasite removal [38,39]. The morning focus on grooming might serve as preparatory stimulation/cleaning for the day ahead, which is not needed in the evening as flies are entering a quiescent phase.

Grooming remains enriched during the day, although this enrichment decreases after the early morning hours. Of the specifically identified grooming states captured by our unsupervised method, we observe that flies spend the most time grooming their fore limbs and eyes, with a lower proportion of time spent in hind grooming and wing grooming. This result is in agreement with a previous study using dust-perturbed flies and optogenetic stimulation that showed a hierarchy to grooming motor programs, with fore grooming prioritized, followed by abdomen grooming, which is captured in our hind grooming state, and finally wing grooming [40]. Unlike this previous work, we demonstrate that the grooming hierarchy occurs during the natural expression of grooming, even when flies are not artificially perturbed by adding dust to their bodies or optogenetically activating specific neurons.

### Flies have longer bouts of proboscis extension during dark hours

We also looked at daily patterns of proboscis extension. While proboscis extension is well correlated with food intake [41], it has also previously been shown to occur during deep sleep [42], and our current analysis does not distinguish between these different types of extension. Previous studies report peak feeding activity centered around lights on and lights off in the mornings and evenings, with more feeding concentrated in the evening [43,44]. Proboscis extension comprises a very small fraction of our data, less than 1% of the overall behaviors across all time points. Because it is such a small component, using compositional data analysis techniques is challenging, as many true zeros exist in the proboscis data which complicate the use of geometric means and log-ratios. To get a better sense of the diurnal nature of proboscis extensions, we instead look at the average duration of proboscis extension bouts over the course of the day (Fig 3D). We find that flies typically stay in the proboscis extension state for about three seconds during night bouts, and about two seconds during day bouts, a difference that may reflect sleeping vs. feeding extensions. By this measure we do not see notable peaks of extensions in the morning and evening, but instead a more general trend of longer bouts at night and shorter bouts during the day. This suggests that flies may be getting different/higher quality sleep during night hours.

### Locomotion speed, in addition to the fraction of time in locomotion, has a daily pattern

Since we can separate specific locomotion bouts from all other time points, we are able to quantify locomotion speed specifically, rather than looking at overall thorax displacement (which includes twitches, aborted attempts are flying, and rocking during abdominal grooming). We find that the diurnal pattern of locomotion speed (the speed of flies when they are in the ‘locomotion’ state, calculated as the mean over a 5 frame rolling window) has peaks around each change in lighting conditions, along with anticipatory increases, particularly for lights on (Fig 3E). This increase in locomotion speed before lights on, and the gradual falling off after lights off, indicates that flies are modulating their movement speed in response to internal cues, rather than only as a startle response or some other reaction to lights on. Depending on where they are in their circadian cycle, flies will move more quickly or slowly. The pattern of locomotion speed does not exactly match the pattern of time spent in locomotion, suggesting that these different aspects of locomotion behavior may have different underlying internal controls.

The daily pattern in locomotion **speed** as well as time spent in locomotion contributes to the activity counts measured in more traditional *D. melanogaster* behavioral assays [5]. In *Drosophila* Activity Monitors, activity counts are recorded each time a fly crosses an infrared beam [9], and these counts could increase due to a combination of increased movement time and increased movement speed. Here, we are able to separate these two factors.

### Flies show behavior and spatial preference changes after Day 3

In addition to daily patterns of behavior, the flies’ behavior changed across days as they weakened and died. Because of the nutritionally incomplete food and the relatively high temperature and low humidity, flies in our experiment all died within 8 days. The behavioral composition remained relatively constant across the first 3 days of the experiment, but starting at Day 4 the idle component began to increase (Fig 1F). This is similar to the increase in the proportion of time male flies spend idle near the end of their lives in a more natural aging paradigm [15].

Flies also show a reduction in the propensity to spend more time near the edge of the arena rather than the center after the first 3 days of the experiment (S8A Fig). The wall-following behavior of *D. melanogaster* likely arises from boundary exploration, possibly as a means of seeking escape from a given enclosure [45]. Over the course of the experiment, flies might decrease wall following behavior as habituation to an unchanging environment decreases exploration activities [46]. However, we also find that flies spend different amounts of time in each of the stereotyped behaviors depending on where they are in the arena (S8B Fig). In particular, flies spend an increased fraction of time in locomotion near the edge of the arena and an increased fraction in idle near the center of the arena. Thus the decline in time spent near the edge of the arena across days could be an artifact of the general increase in time spent in the idle state.

### The unique hour after dawn persists through Day 3

**Fig 4:**
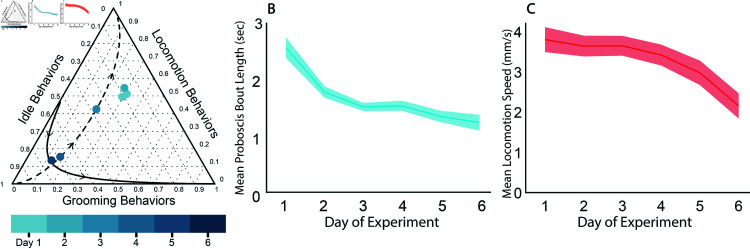
Day wise behavioral changes throughout the experiment. **A** Ternary plot showing the geometric mean of the condensed stereotyped behavioral components of the first hour after lights on across all surviving flies for each complete 24h period. Directions of PC1 (dashed) and PC2 (solid) are also shown, as calculated based on perturbation of the geometric means of all hours from Day 1. **B** Mean proboscis bout length by day of experiment. The shaded region is the standard error. **C** Mean locomotion speed (mm/s) during the stereotyped locomotion state by day of experiment. The shaded region is the standard error.

Since the hour after the lights turn on is such a unique time point in the daily pattern of behavior, we were curious how the behavior components during that hour change over the days of the experiment. The geometric means of the relative proportions of the grooming behaviors, idle behaviors, and locomotion behaviors across all surviving flies in the hour after dawn remain similar for the first 3 days of the experiment, lying in a cluster offset from PC1 in the ternary plot (Fig 4A). Starting at Day 4, however, the hour after dawn components begin falling onto PC1, and are much more similar to other time points. This behavioral composition moves towards lower values of PC1 with age and becomes more similar to the nighttime composition. Thus, as the flies in our experiment weaken and die, not only do their day and night behavior patterns begin to look more similar (Fig 2C), they also lose the distinctive behavioral character of the hour after dawn. As noted previously, the difference between day and night behaviors starts decreasing on Day 2, whereas the hour after dawn remains stable until Day 4. This delay may reflect a difference in the nature or importance of the hour after dawn behavior pattern as opposed the general difference of day vs. night behaviors.

### Proboscis bout length and locomotion speed decline with age

We also asked how proboscis extension bouts and locomotion speed change with age in our experiments. We find that proboscis bout duration decreased steadily through Day 3 and then plateaued (Fig 4B). It has been reported that flies eat more as they age [47], but the limited food source and sub-optimal environmental conditions may change this trend for the flies in our experiment. Additionally, this decrease in bout duration could indicate a decrease in deep sleep in later days, even as time spent in idle increases. In contrast, the average locomotion speed remained steady through Day 3 and then began decreasing with age (Fig 4C). The combination of steady locomotion speed and no increase in the fraction of time spent locomoting means that overall ‘locomotion activity’, comparable to traditional activity counts, does not appreciably change over the first 3 days of the experiment after which there is a decline. Thus here, as in other work, overall activity levels seem to track well with the general health of the flies, and mirror the trend in the change to the behavior components in the first morning hour.

## Conclusion

We report the first measurements of high resolution, fine-grained *D. melanogaster* behavior recorded over many days with high temporal bandwidth. By leveraging recent advances in GPU-based video processing and postural inference, we captured the behavior of freely moving *D. melanogaster* over the course of multiple days, encompassing the behavioral effects of daily rhythms, starvation, aging, and habituation at continuous high resolution. Our data recapitulates many previously described trends in *D. melanogaster* diurnal and aging/dying patterns of behavior, while also finding novel features of the daily and lifelong behavior trends.

The method of behavior capture presented here improves on previous efforts to quantify *D. melanogaster* behaviors over long timescales. Previous studies have succeeded in separating out small and/or peripheral motions from long locomotion bouts and quiescence by using machine vision and supervised behavior classifiers [16,17]. Other work has focused on additional metrics to specifically characterize locomotion activity across days, such as angular velocity or position within an arena [18]. With our combination of postural tracking and unsupervised behavioral classification we are able to specify behaviors at a higher level of granularity, such as different grooming modalities and locomotion states. Our ability to distinguish these fine-grained behaviors allows us to see changes in specific grooming modalities with time of day, and track how flies gradually lose their ability to walk normally with age and stress.

We used our high resolution postural data in combination with fine-grained ethograms to characterize changes in proboscis extension bout duration across time of day, finding an average increased bout duration during night hours. While in this study we cannot distinguish between feeding and sleep associated proboscis extensions, we hypothesize that the increased bout duration during night hours is associated with a deep sleep mode that flies are achieving more frequently at night. As flies age and die in our experiment, their proboscis extension bout length decreases, perhaps revealing an age- or stress-related attenuation of deep sleep.

By investigating locomotion speed specifically during locomotion bouts, we find that locomotion speed, in addition to the fraction of time spent locomoting, has a daily pattern that matches the pattern of activity found in previous circadian studies. It has been found that different aspects of grooming, such as behavior frequency and behavior duration, can be controlled by different mechanisms [16]. While the study presented here does not carry out the necessary genetic and lighting perturbations, it would be interesting to see if locomotion time and locomotion speed are independently controlled mechanisms that together contribute to the typical morning and evening peaks of behavior in *D. melanogaster*.

With compositional data analysis techniques, we identified the hour after lights on as a unique time point in the daily pattern of behavior, with increased levels of all three measured grooming modalities. It is ethologically distinct from the evening hours, possibly due to the difference in the underlying neurogenomic control mechanisms [7,36]. While the overall difference between day and night behaviors decreases constantly across days, the hour after dawn retains a consistent behavior pattern until Day 4. Day 4 is also when other behavior indicators, such as locomotion speed and overall non-idle time begin to decline. In future work, we will investigate how behavior in the hour after dawn is related to overall health, lifespan, and sleep quality in *D. melanogaster*.

Our data addresses several limitations of the high-quality ethological data currently available. Previous work on the temporal structure of behavior has found correlations extending beyond the length of the available high-resolution data, typically 30-60 minutes [48,49]. The data presented here extends these time scales by more than two orders of magnitude. This dataset is also the first to continuously capture high dimensional, high-resolution behavioral data across a diurnal cycle, allowing us to investigate how changes in internal state related to time of day affect behavior. By recording when flies feed (as measured by proboscis extension), this data may also provide new insights into the effects of hunger and satiety. We provide both high-resolution recordings and our postural tracking output to facilitate further data analysis. The analyses presented here leverage only a fraction of the resolution and dimensionality provided by our data, and we hope this 100-fold increase in the amount of high-quality ethological data available will give rise to yet more tools and techniques. Finally, aging/dying in our experiments was significantly accelerated due to nutrient limitation. Future work with new arenas, food sources, and improved environmental control can extend this type of high-resolution behavioral recordings to cover a broad set of use cases across even longer timescales.

## Methods

### Fly rearing

To control for possible genetic effects, we used the highly inbred, ${\text{w}}^{1118}$ wild-type ${\text{iso}}^{KH11}$ strain [50]. ${\text{iso}}^{KH11}$ flies were raised on standard cornmeal media (see github.com/shaevitz-lab/long-timescale-analysis for complete recipe) at 25 ∘C under humidity 60% with a 12-h light/dark cycle, with visible light of  ~ 1300 lux. Before each experiment, we performed egg lays and, on eclosion, flipped flies into new vials. We allowed the flies to age for two days, yielding 2–3 day-old flies, which we anesthetized using ${\text{CO}}_{2}$ and distributed males to arenas to be imaged.

### Media

During experiments, flies were allowed to feed ad lib from a pad of optically transparent media (10% sucrose, 1.5% agarose). We were not able to include a protein source, such as yeast extract, as this led to high levels of fungal growth within 1–2 days that obscured imaging.

### Arena

We constructed experimental arenas out of laser-cut acrylic using acrylic cement (McMaster 7517A4) to adhere layers together (S1B Fig). The bottom layer of each arena consisted of a 3 mm layer of food (described above). Each individual fly was able to freely move about within a 25mm diameter cylinder of height 1.5mm. While we acknowledge that low arena heights may influence behavior, particularly social behaviors which, while not relevant in the current study, might be a topic of future study [51,52], 1.5mm allows common behaviors including locomotion, grooming, and proboscis extensions [53,54] and drastically reduces the time spent overturned and on the walls. Because these arenas have straight walls, flies are able to walk along the sides, which can cause limb occlusions that pose difficulties to downstream postural tracking. To address this, we used a low arena height that impedes flies from easily maneuvering off the base layer. We also coated the top and walls with Sigmacote (Sigma-Aldrich SL2), which discourages flies from walking on the ceiling of the arena but does not fully restrict them from walking on the edges of the arenas.

### Imaging and illumination

The arenas are lit from above using 880nm IR LED pads (Advanced Illumination BL040801-880-IC). Below each arena, we placed high-resolution, high frame-rate cameras (FLIR BFS-U3-32S4M-C) paired with 880nm band-pass filters (ThorLabs FB880-70) (S1 Fig A). This combination allows bright, uniform lighting across the arenas permitting extremely short exposure times to reduce motion blur. Imaging from above and recording from below also eliminates condensation in the arenas. We found that the ideal balance between contrast and motion blur was at 1 ms exposure time. In addition, we used a pair of visible light LED panels at the top of the tent enclosing the experimental setup to provide a 12-h visible light ( ~ 6500 lux) and 12-h darkness cycle (<6 lux), matching the timing of the light/dark cycle under which experimental flies were reared. This visible light cycle did not appreciably affect the IR imaging.

### Temperature and humidity

We recorded temperature and humidity within the imaging enclosure throughout the trials (S2 Fig) with an Extech RHT10 datalogger. As temperature and humidity have known effects on fly behavior [55,56], these data are provided with the behavioral dataset so that they may be taken into account (S9 Fig). The environmental controls of the room in which our experiments were housed cycle on and off, leading to ~1 ∘ C temperature fluctuations with a period of  ~ 1 h.

### Acquisition software

We used a modified version of campy (github.com/Wolfffff/campy) forked from github.com/ ksseverson57/campy which was developed by Kyle Severson. We altered the package to suit our specific use-case, including chunking videos and adjusting the exception handling. Campy pipes frames from FLIR’s Spinnaker SDK (PySpin) to FFmpeg. The flexibility of FFmpeg allows us to drastically reduce the file size of our videos by utilizing hardware-based compression. Specifically, we use Nvidia NVENC (hevc_nvenc) paired with the segment_time flag to produce hour-long chunks. This increased compression makes it feasible to perform high-throughput recordings of 8 flies simultaneously on a single computer. To facilitate ease of use in analysis and distribution, we merge these videos into long videos; however, because loading tens of millions of frames and instances can cause IO issues, we use hour long segments for training.

The machines used for recording were running Windows 10 with 64GB RAM, Intel Core i7-8700K CPU processor, and either Nvidia Quadro RTX 4000, Quadro P2000, or GeForce RTX 2080 GPU.

### SLEAP tracking

After imaging, SLEAP [19] was used to estimate the pose of each individual and maintain identity across videos. We used a 14 node skeleton: head, eyes (eyeL, eyeR), proboscis, thorax, abdomen, fore legs (forelegL, forelegR), mid legs (midlegL, midlegR), and the hind legs (hindlegL, hindlegR). We labeled 1930 individuals across 482 frames. 434 frames (1738 instances) were used for training, with 48 frames (192 instances) reserved for validation. We trained a U-Net based model with a receptive field size of 76 pixels ($2.\bar{6}$mm) on Nvidia A100 GPUs, achieving a mean localization error of less than .1mm (S2 Fig). The complete hyperparameter set is provided along with the model. We include some training data from recordings not included in the final dataset due to early truncation but with identical frame rates and resolution. To facilitate dealing with the more than 500 million frame dataset, we use SLURM to distribute our inference across 30 Nvidia P100 GPUs at approximately 20 fps yielding approximately 600fps – 6x speed – tracking. After inferring locations with identity, we merged the resulting .slp files together and ran SLEAP’s identity tracking script to preserve identity over time. For convenience of analysis and storage, we convert each .slp file to HDF5. Since individuals are in separate chambers, we can validate these identity tracks by the amount of time spent in each quadrant of the arena. The pipeline for sectioning, merging, and tracking can be found on the associated GitHub repository.

### Edge detection

While flies spend the majority of their time in the flat bottoms of the arenas, there is a small proportion of time ( ~ 5%) when they are oriented sideways with respect to the cameras with their tarsi on the walls of the arenas. In this position the legs are often occluded and difficult to identify, leading to SLEAP tracking errors. In order to provide a flag for time points when the flies are on the edge and tracking fidelity is compromised we used the MATLAB Classification Learner App to train an SVM to identify whether flies are on or off the edge based on the all-by-all distances between tracked body coordinates (excluding the proboscis), the speed of each body coordinate, and the distance between each body coordinate and the edge of the arena. We used 2788 training points equally split between on and off edge instances, and sampled evenly across all experimental flies. Our final model accurately labeled 95% of held out validation points (S3 Fig).

### Unsupervised behavioral classification

To identify stereotyped behaviors from body-part dynamics, we adapted the previously described MotionMapper pipeline [11] for our data (S4 Fig). We first partially filled in missing data, interpolating all missing data for head and thorax points using Piecewise Cubic Hermite Interpolating Polynomial (PCHIP), to allow for subsequent egocentrizing. For all other nodes, we performed PCHIP interpolation with a limit of filling 5 consecutive missing values. Further, for the proboscis node, we replaced all missing values with the location of the head, representing a retracted proboscis. Further, we performed a median filter on all nodes with a window size of 5 and Gaussian filtering with standard deviation 1 and window size 5. Following this, we egocentrized the data by shifting all individuals so that the thorax is at (0, 0) and rotating each node location so that the thorax-head connection falls along the positive x-axis. After this, we calculated the Lomb-Scargle periodogram on rolling windows for each coordinate of each node. Because the Lomb-Scargle periodogram allows the utilization of unevenly sampled data and avoids the necessity of providing fully interpolated data it is particularly useful for postural tracking data. Further, by adjusting the window size based on our frequency of interest, we are able to capture behaviors across timescales similar to the envelope size in continuous wavelet transforms.

We compiled a representative subsample of our data by selecting 141 fly hours evenly across flies and time of day. Because flies are dying throughout the course of the experiment our sample set is slightly skewed towards earlier days to maintain even sampling across flies. We filtered training points from this subsample of data by removing time points where the flies were on the edge. We also removed time points we classified as idle where the total amplitude of the wavelets was less than $0.5012mm^{2}$, a threshold we empirically determined to separate the majority of idle instances where the fly was largely motionless. From these, we sampled 36000, or the maximum number of unfiltered time points, from each fly-hour. From each of the these groups, we importance sampled 454 time points for a total of 64,014 training points.

We embedded our importance-sampled training set into two dimensions using UMAP and used this map for behavioral segmentation (S5 Fig). We found that UMAP resulted in superior separation into unique clusters for the total training set when compared with t-SNE. We used kernel density estimation to create a 2D probability distribution of our training points. To identify distinct peaks in the density of training points we eliminated points of extreme low density and utilized adaptive thresholding on the resulting distribution. We adjusted parameters by eye to achieve distinct clusters for obviously separate peaks of density while aiming to avoid oversegmentation.

In order to assign specific discrete behaviors to each region of stereotyped power spectra we randomly selected clips from our sample set (141 fly hours) corresponding to each region. We imposed a minimum duration based on the dwell time distribution for each region to avoid very short bouts where behaviors might be difficult to identify. We identified six well-defined stereotyped behaviors (proboscis extension, fore grooming, hind grooming, wing grooming, altered locomotion, and locomotion) as well as many clusters that corresponded to idle behaviors with single-joint SLEAP tracking errors.

We then embedded our entire dataset into the same two dimensional space. Using the boundaries defined on the training set we assigned all time points to one of our six well-defined stereotyped behaviors, idle, edge (as called by our edge detector), or unstereotyped. With this method, only  ~ 15% of our data is classified as unstereotyped behavior.

Dwell times within these behavior states can vary from single frames to many hundreds of frames. To identify a reasonable minimum bout length we fit two geometric distributions to the total dwell time histogram. We selected 5 frames ( ~ 1/20 of a second) as a minimum bout duration, as this excludes  ~ 95% of bouts from the distribution dominated by shorter bouts, and only  ~ 14% of bouts from the distribution of longer bouts, which we take to include legitimate behavior bouts. We forward-filled ethograms with this bout duration, assigning any bout of 4 frames or less to the previous behavior of long duration.

### Principal Components Analysis

Using the compositions package in R [28] we carried out robust principal components analysis across all fly-hours with the Minimum Covariance Determinant (MCD) method. In brief, 7-component fly-hours are converted into 6 linearly independent components using and isometric log-ratio transform, which permits the use of statistical analysis tools relying on real numbers in an orthonormal basis. Principal components analysis is carried out on these 6-component transformed fly-hours, using the MCD method to calculate covariance in a way that is robust to statistical outliers. The result is then backtransformed into 7 centered log-ratio transformed components, which gives loadings that have interpretable meanings related to the original behavioral components.

The first two principle components, which explain  ~ 75% of the variance across fly hours are analyzed above. PC3 largely separates the first two experiments (begun 02/17/2022 and 03/13/2022) from the second set of experiments (begun 03/26/2022 and 04/18/2022) (S7 Fig) which took place at higher temperatures (S9 Fig). The difference in the projections of each fly-hour along PC3 between the two sets of experiments is lowest on Day 1, and increases over experimental days.

## Supporting information

S1 Table Metadata table outlining the data collected. (PDF)

S1 Fig Schematic of imaging setup and arena design. (PDF)

S2 Fig Prediction error plot. (PDF)

S3 Fig Illustration of edge detection method. (PDF)

S4 Fig Schematic of behavioral classification pipeline. (PDF)

S5 Fig Plot showing the density map of 2D embedding values from UMAP along with the region assignments. (PDF)

S6 Fig Line plot showing the cumulative variance explained by PCs of behavioral components on the complete data set. (PDF)

S7 Fig Box plots showing PC3 of behavioral components PCA. (PDF)

S8 Fig Behavioral characteristics by radial position and radial position distributions by day of experiment. (PDF)

S9 Fig Line plots showing temperature and humidity measured throughout the experiments. (PDF)
