# Supplementary material for: Capturing continuous, long timescale behavioral changes in Drosophila melanogaster postural data
Source: PLoS Comput Biol. 2025 Feb 3;21(2):e1012753. doi: 10.1371/journal.pcbi.1012753 (PMC11813078; doi:10.1371/journal.pcbi.1012753)
Supplement: S1 Table — (PDF) [file pcbi.1012753.s001.pdf]

Date	Experiment	Camera	Start Time (UTC)	File
2/17/2022	exp1	Camera 1	2022/02/17 17:37:18	20220217-lts-cam1
2/17/2022	exp1	Camera 2	2022/02/17 17:37:18	20220217-lts-cam2
2/17/2022	exp1	Camera 3	2022/02/17 17:46:39	20220217-lts-cam3
2/17/2022	exp1	Camera 4	2022/02/17 17:46:39	20220217-lts-cam4
3/13/2022	exp2	Camera 3	2022/03/13 02:14:15	20220313-lts-cam3
3/13/2022	exp2	Camera 4	2022/03/13 02:14:15	20220313-lts-cam4
3/26/2022	exp3	Camera 3	2022/03/26 19:50:45	20220326-lts-cam3
3/26/2022	exp3	Camera 4	2022/03/26 19:50:45	20220326-lts-cam4
4/18/2022	exp4	Camera 1	2022/04/18 19:06:00	20220418-lts-cam1
4/18/2022	exp4	Camera 2	2022/04/18 19:06:00	20220418-lts-cam2
4/18/2022	exp4	Camera 3	2022/04/18 19:07:28	20220418-lts-cam3
4/18/2022	exp4	Camera 4	2022/04/18 19:07:28	20220418-lts-cam4

S 1Table. Metadata table outlining the data collected. Each row represents a single camera recording on a single day covering 4 wells. Here, we show the composition of experiments starting from mid day to evening. The provided File column corresponds to the H5 file containing tracks of each recording. Complete metadata at the per-fly level is provided in the associated data repository.
