# Supplementary material for: Capturing continuous, long timescale behavioral changes in Drosophila melanogaster postural data
Source: PLoS Comput Biol. 2025 Feb 3;21(2):e1012753. doi: 10.1371/journal.pcbi.1012753 (PMC11813078; doi:10.1371/journal.pcbi.1012753)
Supplement: S1 Fig — (PDF) [file pcbi.1012753.s002.pdf]

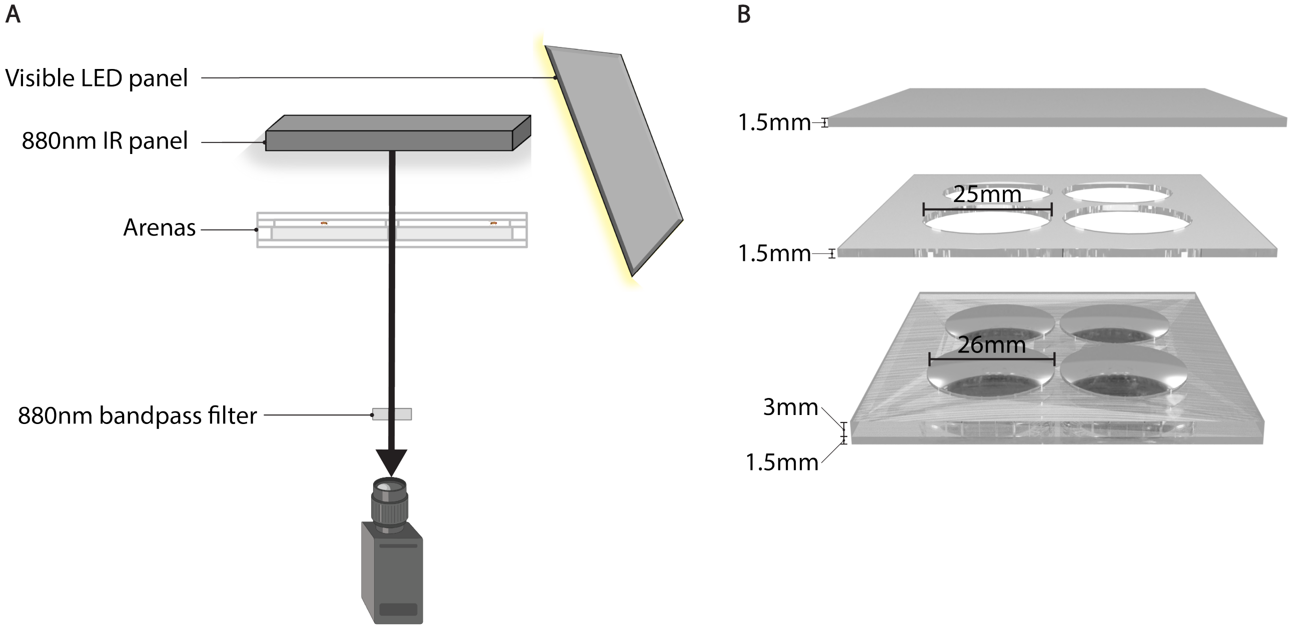

S1 Fig. A Schematic of imaging setup. Experimental arenas were illuminated from above with 880nm LED pads to permit constant recording and two visible LED panels (only one shown) on a 12-hour light/dark cycle. Flies were recorded from below using 880nm bandpass filters on each camera to ensure uniformity across visible light changes. **B** Experimental arena schematic. Arenas were constructed from layers of transparent laser-cut acrylic, with a 3mm deep pad of sucrose-agarose media beneath a 1.5mm deep chamber enclosed by a solid layer of acrylic. Arena layers were held together by lab tape, which prevented escape while also permitting airflow. This figure was created in part with biorender.com
