# Supplementary material for: Capturing continuous, long timescale behavioral changes in Drosophila melanogaster postural data
Source: PLoS Comput Biol. 2025 Feb 3;21(2):e1012753. doi: 10.1371/journal.pcbi.1012753 (PMC11813078; doi:10.1371/journal.pcbi.1012753)
Supplement: S2 Fig — (PDF) [file pcbi.1012753.s003.pdf]

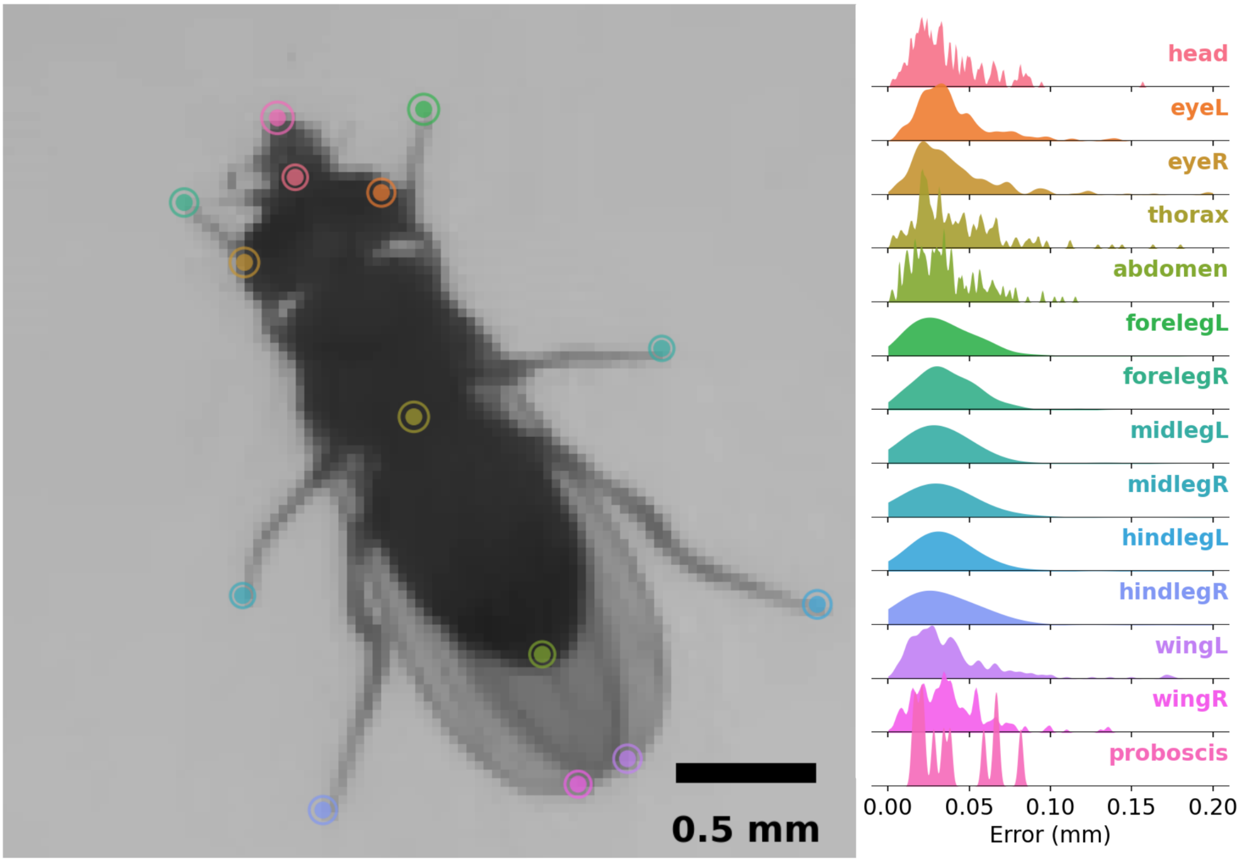

S2 Fig. Prediction error plot. The average error distance is approximately 2.22px, corresponding to 78.5µm. Our model's mean average precision (mAP) is 0.70, and the error distance 95th percentile is 4.14px. More metrics and complete models are available in the main dataset.
