# Supplementary material for: Capturing continuous, long timescale behavioral changes in Drosophila melanogaster postural data
Source: PLoS Comput Biol. 2025 Feb 3;21(2):e1012753. doi: 10.1371/journal.pcbi.1012753 (PMC11813078; doi:10.1371/journal.pcbi.1012753)
Supplement: S3 Fig — (PDF) [file pcbi.1012753.s004.pdf]

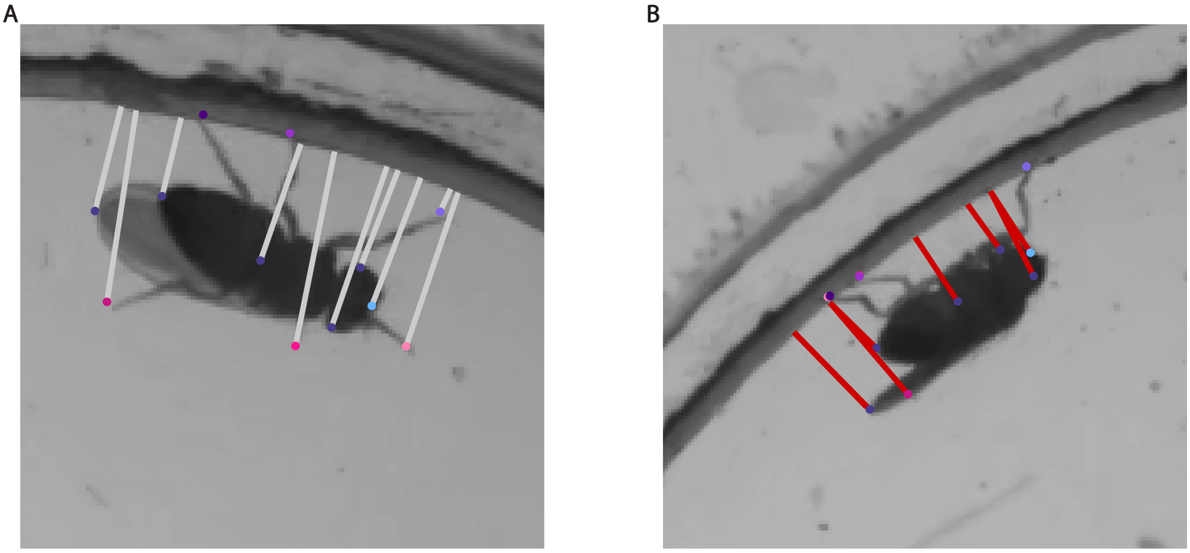

S3 Fig. Illustration of edge detection method. An SVM classifier uses the all-by-all distances between all body part coordinates, except for the proboscis, the speed of each body part, and the distance for each body part from the edge to classify time points as ’off edge’ (example shown on left) or ’on edge’ (example shown on right). Points directly on the edge, such as some of the tarsi in these images, have an edge distance of 0, which naturally cannot be shown.
