# Supplementary material for: Capturing continuous, long timescale behavioral changes in Drosophila melanogaster postural data
Source: PLoS Comput Biol. 2025 Feb 3;21(2):e1012753. doi: 10.1371/journal.pcbi.1012753 (PMC11813078; doi:10.1371/journal.pcbi.1012753)
Supplement: S4 Fig — (PDF) [file pcbi.1012753.s005.pdf]

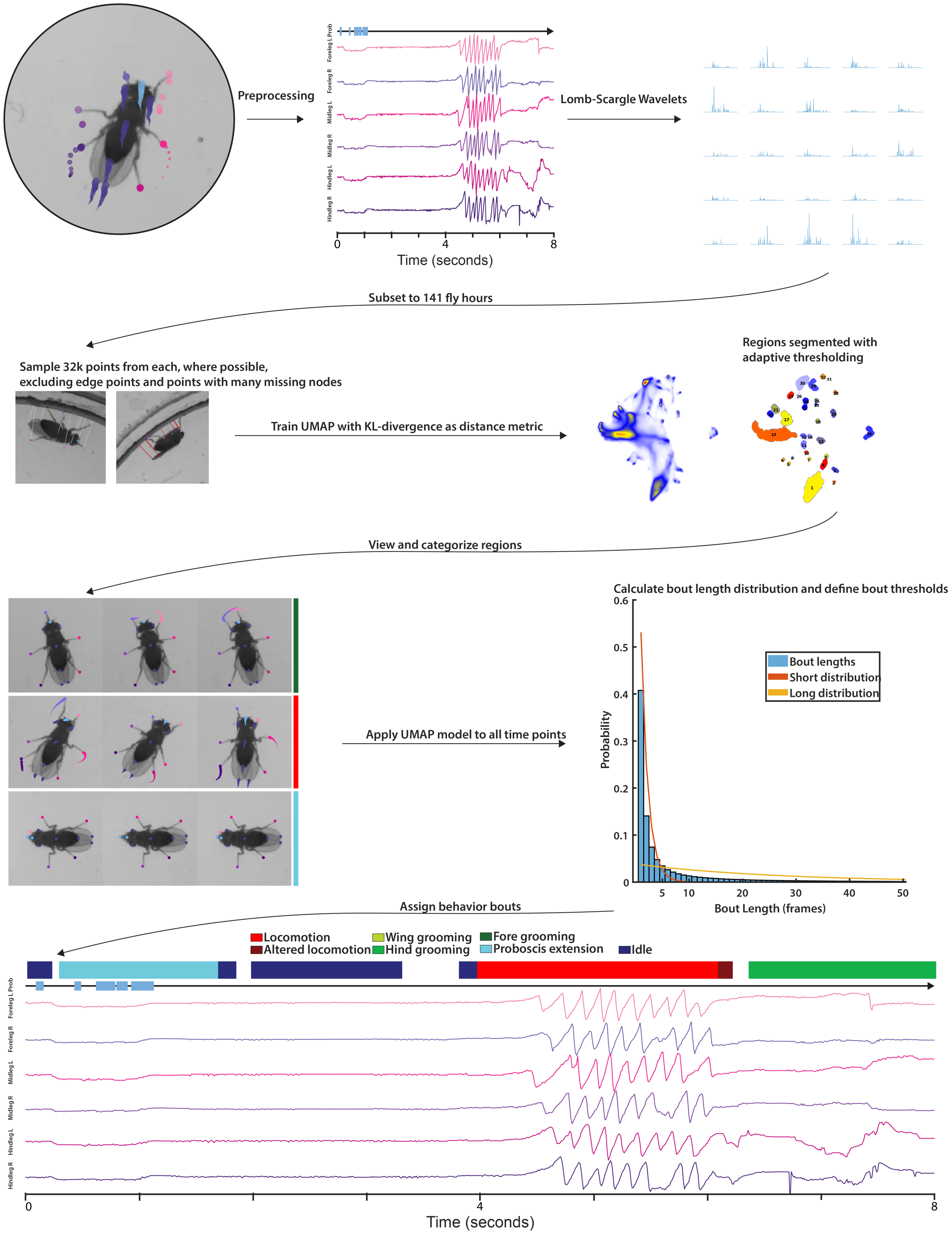

S4 Fig. Schematic of behavioral classification pipeline. The behavioral pipeline shows the flow of data from pose estimation from SLEAP through behavioral bout assignment.
