# Supplementary material for: Capturing continuous, long timescale behavioral changes in Drosophila melanogaster postural data
Source: PLoS Comput Biol. 2025 Feb 3;21(2):e1012753. doi: 10.1371/journal.pcbi.1012753 (PMC11813078; doi:10.1371/journal.pcbi.1012753)
Supplement: S5 Fig — (PDF) [file pcbi.1012753.s006.pdf]

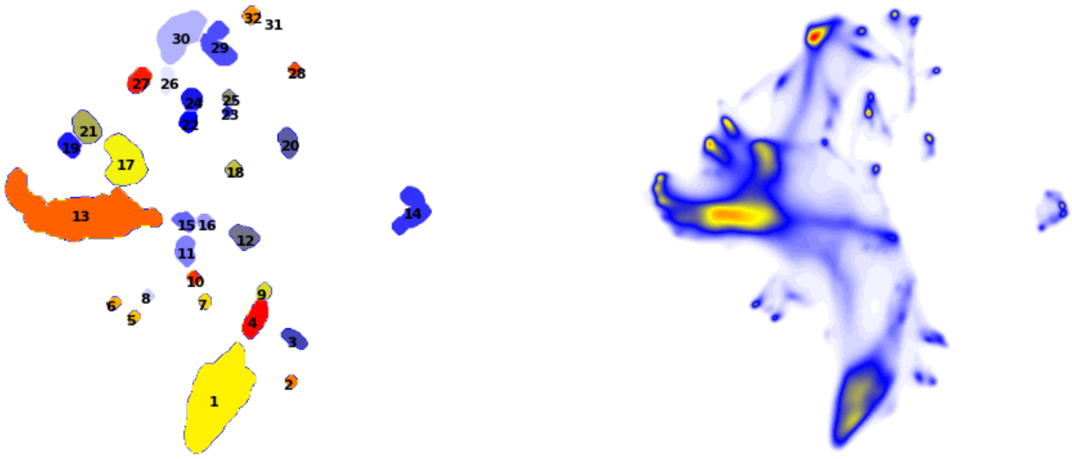

S5 Fig. Plot showing the density map of 2D embedding values from UMAP along with the region assignments. Regions 2, 3, 4, 5, 9, 12, 15, 16, 18, 20, 23, 25, 28, 31, and 32 were assigned to idle behavior, 14 was assigned to proboscis extension. Regions 1, 6, 7, 8, 10, and 11 were assigned to foreleg grooming. Regions 26, 27, and 30 were assigned to hind grooming. Regions 22, 24, and 29 were assigned to wing grooming behavior. Regions 17, 19, and 21 were assigned to altered locomotion. Finally, region 13 was assigned to locomotion.
