# Supplementary material for: Capturing continuous, long timescale behavioral changes in Drosophila melanogaster postural data
Source: PLoS Comput Biol. 2025 Feb 3;21(2):e1012753. doi: 10.1371/journal.pcbi.1012753 (PMC11813078; doi:10.1371/journal.pcbi.1012753)
Supplement: S6 Fig — (PDF) [file pcbi.1012753.s007.pdf]

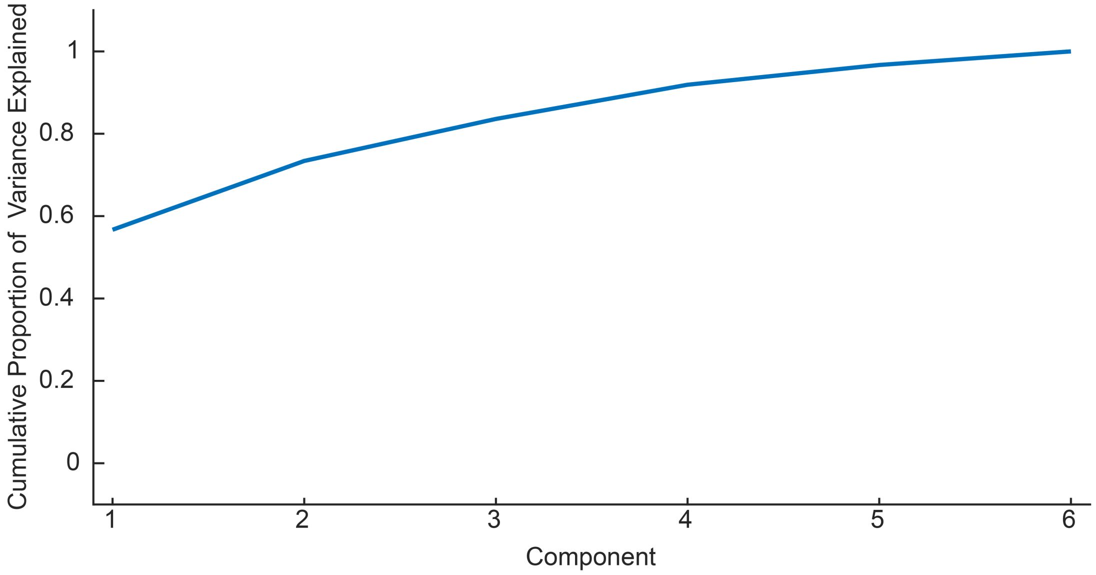

S6 Fig. Line plot showing the cumulative variance explained by PCs of behavioral components on the complete data set.
