# Supplementary material for: Capturing continuous, long timescale behavioral changes in Drosophila melanogaster postural data
Source: PLoS Comput Biol. 2025 Feb 3;21(2):e1012753. doi: 10.1371/journal.pcbi.1012753 (PMC11813078; doi:10.1371/journal.pcbi.1012753)
Supplement: S7 Fig — (PDF) [file pcbi.1012753.s008.pdf]

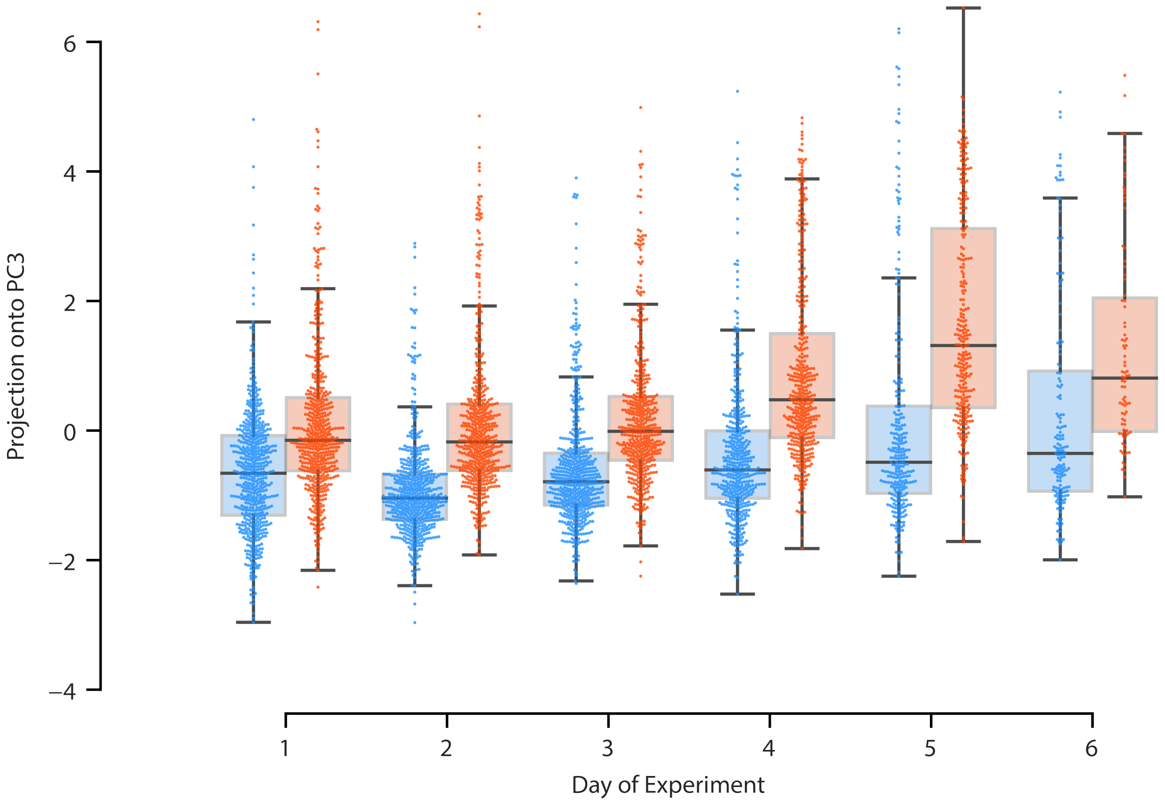

S7 Fig. Box plots showing PC3 of behavioral components PCA. Experimental group 1 (experiments 1 and 2) is shown in blue and experimental group 2 (experiments 3 and 4) are shown in red. PC3 separates our experimental groups and this separation becomes more significant farther into the experiments.
