# Supplementary material for: Capturing continuous, long timescale behavioral changes in Drosophila melanogaster postural data
Source: PLoS Comput Biol. 2025 Feb 3;21(2):e1012753. doi: 10.1371/journal.pcbi.1012753 (PMC11813078; doi:10.1371/journal.pcbi.1012753)
Supplement: S8 Fig — (PDF) [file pcbi.1012753.s009.pdf]

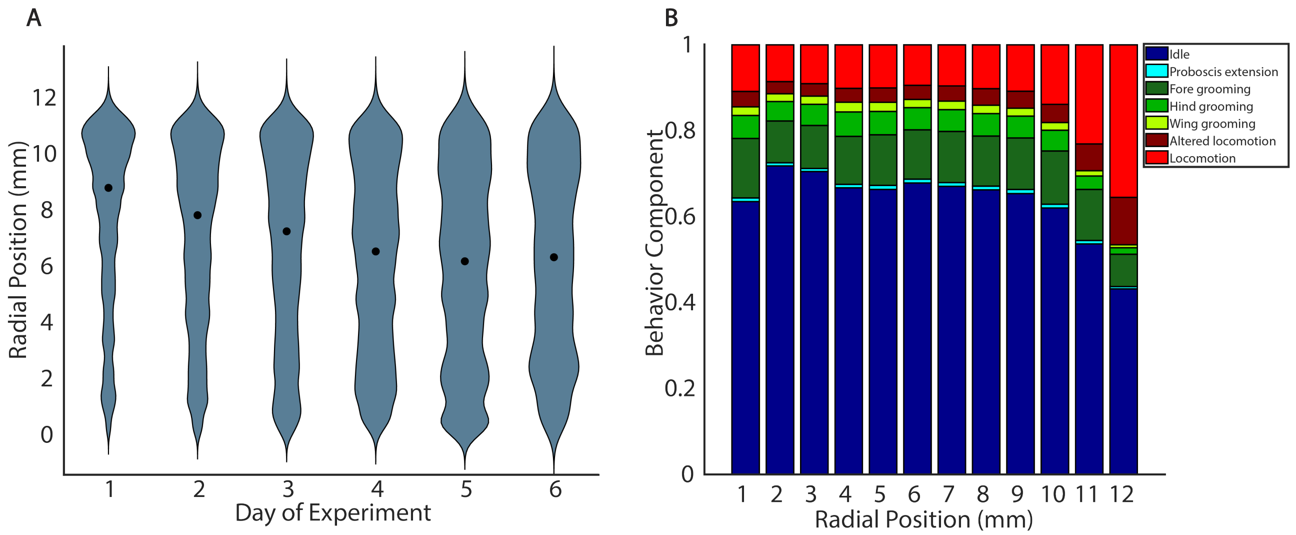

S8 Fig. Behavioral characteristics by radial position and radial position distributions by day of experiment. **A** Violin plot showing the distributions of radial position by day of experiment. **B** Barplot of behavioral components by radial position.
