# Supplementary material for: Capturing continuous, long timescale behavioral changes in Drosophila melanogaster postural data
Source: PLoS Comput Biol. 2025 Feb 3;21(2):e1012753. doi: 10.1371/journal.pcbi.1012753 (PMC11813078; doi:10.1371/journal.pcbi.1012753)
Supplement: S9 Fig — (PDF) [file pcbi.1012753.s010.pdf]

A

B

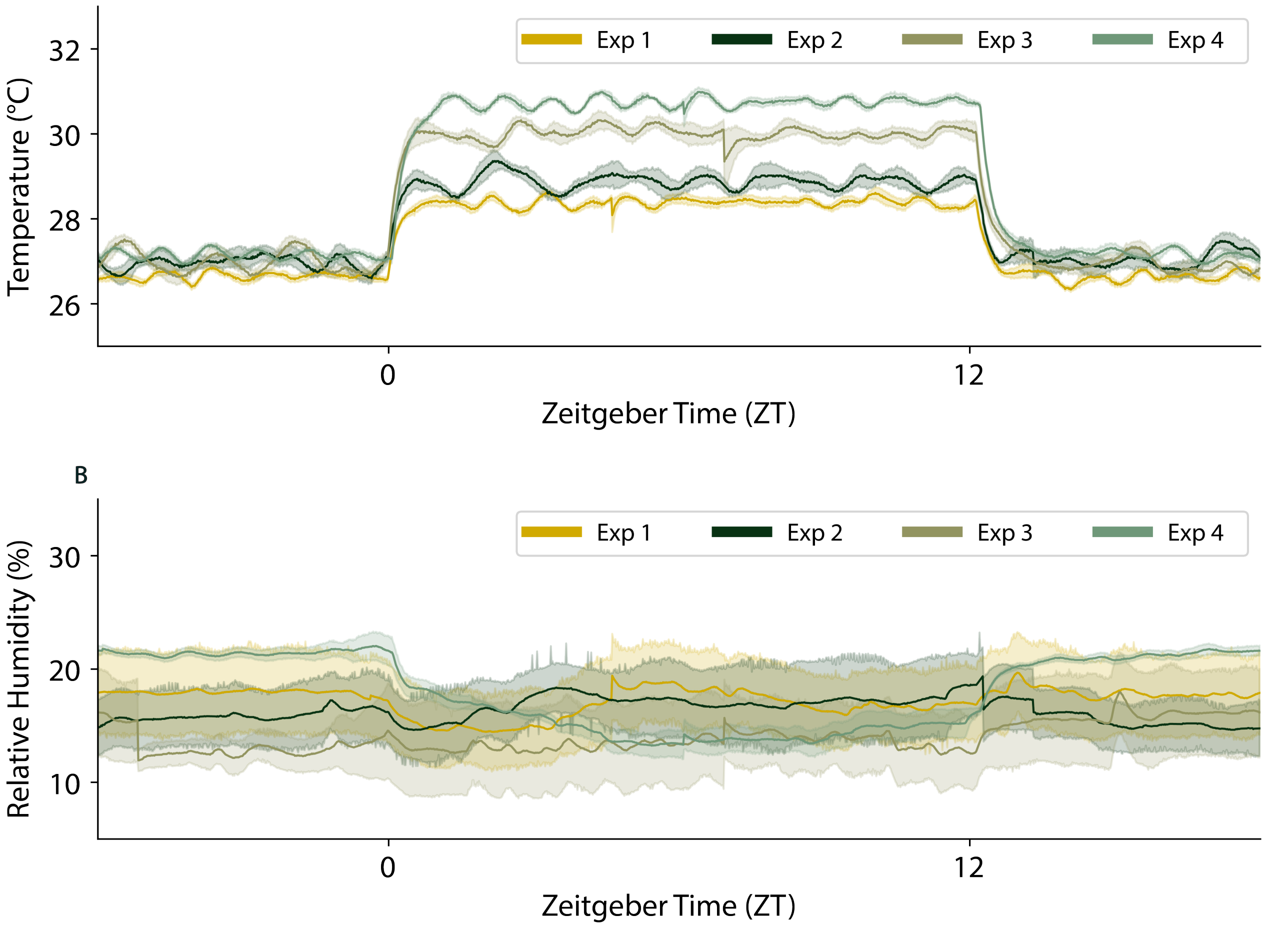

S9 Fig. Line plots showing temperature and humidity measured throughout the experiments. All measurements were taken a 1 minute intervals and have been trimmed to only include points where at least one fly in the experiment is alive. **A** Line plot showing the variation in temperature across experiments and zeitgeber time. The shaded region is the standard error. **B** Line plot showing the variation in humidity across experiments and zeitgeber time. The shaded region is the standard error.
